# Takotsubo Cardiomyopathy as a Manifestation of Dysautonomia in Guillain-Barré Syndrome: A Case Series and Review of the Literature

**DOI:** 10.7759/cureus.16069

**Published:** 2021-06-30

**Authors:** Dyanet Puentes, Daniela Teijelo, Tamara S Stiep, Sishir Mannava, Jason Margolesky

**Affiliations:** 1 Neurology, University of Miami, Miami, USA

**Keywords:** all neurology, guillain-barré syndrome, dysautonomia, takotsubo cardiomyopathy

## Abstract

Guillain-Barré syndrome (GBS) is an inflammatory polyneuropathy that classically presents with low back pain, sensory paresthesias, and rapidly progressive weakness. Patients with GBS can develop dysautonomia, and Takotsubo cardiomyopathy (TCM) is a rare potential manifestation of this dysautonomia. This association has been reported only 12 times in the literature so far, which we review here. We present two cases of GBS associated with TCM, to increase awareness with regard to this comorbid relationship, which would encourage prompt initiation of proper supportive care to avoid morbidity and mortality.

We report the case of two patients - a 58-year-old man and a 79-year-old woman - who developed TCM in the setting of axonal variants of GBS. Electrodiagnostic results, cerebrospinal fluid profiles, and echocardiogram findings were consistent with these diagnoses. Both patients were treated with intravenous immunoglobulin (IVIG) in an intensive care unit (ICU) setting. Echocardiogram findings were reversible.

TCM should be recognized as a potential complication of GBS in patients with dysautonomia. This case series adds to the sparse body of literature describing the association between these two conditions. It is not clear if patients with axonal variants of GBS are more predisposed to developing TCM; further, larger case series in the future may help identify the risk factors associated with it. We hope to shed more light on this possible association to expedite the diagnosis and management of this condition.

## Introduction

Guillain-Barré syndrome (GBS), also referred to as acute inflammatory demyelinating polyneuropathy (AIDP), is an inflammatory polyneuropathy classically presenting with low back pain, sensory paresthesias, and rapidly progressive weakness. Symptoms are often encountered after an infectious trigger, and clinical findings are thought to be caused by autoimmune-mediated damage to peripheral nerves and their spinal roots leading to demyelination [1]. Sensory-only variants and less typical distributions of weakness are also encountered, and, in rare cases, patients can present with axonal subtypes of GBS, such as acute motor axonal neuropathy (AMAN) and acute motor and sensory axonal neuropathy (AMSAN), which account for approximately 5% of GBS cases in North America and Europe [2].

Takotsubo cardiomyopathy (TCM) is a rare, potentially fatal manifestation of dysautonomia associated with GBS; there are only 12 previously reported cases of this association, which we summarize in Table 1. The exact mechanism by which GBS and TCM are related still remains unclear, but it is hypothesized that the combination of dysregulated autonomic tone with an exaggerated sympathetic surge that is known to occur in GBS triggers this myocardial stress response [3,4]. In light of the dearth of existing literature on this association, we present two cases of GBS associated with TCM, in order to increase awareness about this comorbid relationship, which would hopefully lead to prompt initiation of proper supportive care and avoid morbidity and mortality.

## Case presentation

Case 1

**Figure 1:**
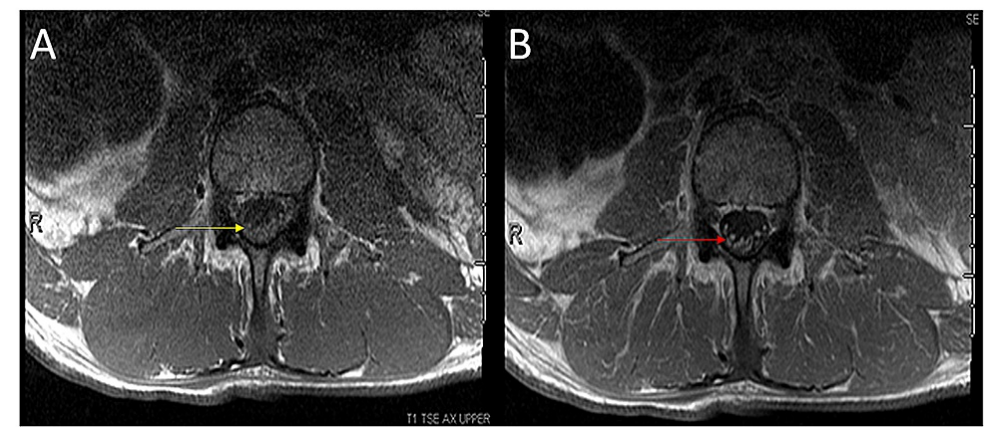
MRI lumbar spine with and without gadolinium A. MRI lumbar spine T1 axial image with unenhanced nerve roots (yellow arrow). B. MRI lumbar spine T1 post-gadolinium axial image with the enhancement of ventral nerve of the cauda equina (red arrow) MRI: magnetic resonance imaging

A 58-year-old man presented with progressive generalized weakness following a viral illness, requiring emergent intubation. Lumbar puncture, electromyogram/nerve conduction studies (EMG/NCS), and MRI imaging (Figure 1) were consistent with the diagnosis of axonal-variant GBS. The cerebrospinal fluid profile revealed albuminocytologic dissociation with 2 cells/mm^3 ^and protein of 93 mg/dl. EMG/NCS was diagnostic for severe sensorimotor polyneuropathy with axonal and demyelinating features. At the nadir of his weakness, the patient was areflexic with flaccid paralysis below his neck [Medical Research Council (MRC) grade 0/5 in all extremities], but with intact extraocular movements. He developed severe dysautonomia, including labile blood pressure and persistent tachycardia, and a transthoracic echocardiogram (TTE) revealed a severely reduced ejection fraction (EF) of 15% with mild to moderate lateral and anterior wall hypokinesis, consistent with TCM. The patient was initially treated with 2 g/kg intravenous immunoglobulin (IVIG) and subsequently achieved improved motor strength allowing for acute rehabilitation as well as complete resolution of TCM on TTE. Six weeks after the IVIG treatment, he was showing antigravity in both lower extremities with near-normal strength in his upper extremities.

Case 2

A 79-year-old woman was transferred from El Salvador with a four-week history of progressive weakness with acute decline leading to respiratory failure. EMG/NCS was consistent with the AMSAN variant of GBS, with reduced amplitude of sampled sensory nerve action potentials and compound motor action potentials with relatively spared conduction velocities. At the nadir of her weakness, proximal upper extremity muscles were scored 0/5 but with 2/5 finger movements, in the lower extremities, proximal muscles were scored 1/5 but with 3/5 dorsiflexion and plantar flexion. TTE revealed moderately reduced EF of 30-35% with hypokinesis of the middle segments of the left ventricle, with hyperdynamic basal and apical segments, consistent with TCM. The echocardiogram normalized but the patient had minimal improvement in motor strength (MRC grade improvement of 1 point in wrist extension and foot dorsiflexion bilaterally) at six weeks following IVIG.

## Discussion

These patients had severe axonal variants of GBS with profound weakness, respiratory failure, and dysautonomia. We consider TCM to be a manifestation of this dysautonomia. In GBS, the development of dysautonomia may be attributed to the involvement of small nerve fibers, specifically the afferent nerves from baroreceptors, the parasympathetic system efferent fibers that supply the heart, and the sympathetic fibers that regulate vasomotor and sweat gland stimulation function [2,5]. Up to two-thirds of patients with GBS may experience dysautonomia [6]. Dysautonomia may present as blood pressure fluctuations, arrhythmias, heart rate disturbances, transient cardiomyopathy, left ventricular dysfunction, cardiac arrest, gastrointestinal dysmotility, pupillary dysfunction, urinary retention, fevers, or hypothermia [7]. The treatment for this autonomic dysfunction is usually supportive. Dysautonomia is prevalent in GBS and is the most common cause of sudden death in these individuals [8], highlighting the importance of its recognition, even though the prevalence of TCM in these individuals is not known.

To our knowledge, there have only been 12 prior case reports describing the association between TCM and GBS [3,5,6,9-17]. We searched PubMed with the keywords "Guillain Barre Syndrome," "acute inflammatory demyelinating polyradiculoneuropathy," and "Takotsubo Cardiomyopathy" in order to find prior case reports. Including our two cases, 12 of the 14 patients were female. Elevated cardiac enzymes were reported in 12 of 14 cases (levels not reported in one case). Most cases reported concomitant dysautonomia symptoms in addition to TCM and most required mechanical ventilation. When available, we extracted the following data from these reports: age, sex, electrodiagnostic results, cerebrospinal fluid profiles, echocardiogram findings, EKG results, GBS treatment, and outcomes, which are summarized in Table 1. Additionally, one case of TCM was reported in a 51-year-old woman with Miller-Fisher syndrome [18].

**Table 1:** Summary of GBS and TCM cases GBS: Guillain-Barré syndrome; TCM: Takotsubo cardiomyopathy; EMG: electromyogram; NCS: nerve conduction studies; CSF: cerebrospinal fluid; EKG: electrocardiogram; AMAN: acute motor axonal neuropathy; PMNL: polymorphonuclear leukocytes; LVEF: left ventricular ejection fraction; IVIG: intravenous immunoglobulin; PLEX: plasma exchange; NR: not reported

Case	Age in years/sex	EMG/NCS	CSF	2D echo	EKG	Treatment	Outcome (time to recovery)
1 [15]	77/M	"Completely silent electrical activities for both compound muscle action potentials and sensory nerve action potentials, except for the left median nerve compound muscle action potentials"	Cell count: 0/mm^3^; protein: 73 mg/dl	NR	T wave inversion in leads II, III, aVF, and V1-V6	PLEX and IVIG x 5 days	Deceased from complications
2[11]	69/F	Axonal sensorimotor polyneuropathy	No albuminocytologic dissociation	"Left ventricular inferior wall and apical akinesia and decreased left ventricular ejection fraction"	T wave inversion and ST-segment elevation in anterolateral	IVIG x 5 days	1 month
3 [6]	44/F	NR	NR	"Extensive global dyskinesia of left ventricle with hypokinesia and ballooning of apex and hyperkinesia of the base. EF of 12%"	T wave inversion	NR	2-4 weeks
4 [13]	60/F	"Reduced motor conduction velocity in all limbs. Sensory conduction was normal in the lower limbs. Slight reduction in the recruitment pattern during effort"	Total protein concentration of 91 mg/dl and cell count <1/mm^3^	Apical akinesis with basal function preserved and LVEF of 45%	Diffuse negative T waves	IVIG x 5 days	2 weeks
5 [5]	68/F	"Consistent with GBS"	"Consistent with GBS"	EF of 25% with severe apical hypokinesis	T wave inversion and ST elevation	PLEX	2 months
6 [12]	70/F	"Consistent with GBS"	NR	LVEF of 30% with a severe hypokinetic anterior septum and left ventricular apex	Negative for ischemic changes	IVIG	4 months
7 [3]	39/F	NR	NR	Akinesia of the septum and inferior left ventricular wall and apical akinesis LVEF of 10%	Sinus tachycardia with nonspecific ST-T segment changes	NR	4 months
8 [10]	65/F	"Temporal dispersion, significantly slow conduction velocities, prolonged distal and F wave latencies, and abnormal upper extremity sensory nerve conduction"	Elevated protein (450 mg/L) with normal cells (2/mm^3^)	Dilated and severe hypokinetic LVEF of 20%	Sinus tachycardia with nonspecific ST-T segment changes; repeat EKG: inverted T waves	IVIG x 5 days	1 month: normal EKG, LVEF; still with heavy peripheral, symmetrical, and especially motor polyneuropathy
9 [14]	59/F	"Consistent with GBS"	"Consistent with diagnosis of GBS"	Apical and mild ventricular akinesis with preserved wall motion of left ventricular base LVEF of 25%	ST elevation in precordial leads	IVIG x 5 days	NR
10 [12]	27/F	"Severe neurogenic process affecting the motor nerves of all 4 extremities, with absent motor-evoked responses. Sensory evoked response amplitude normal in left superficial fibular, sural, median, and ulnar nerves" - AMAN	0 RBCs, 14 WBCs (95% lymphocytes, 2% PMNL, 3% monocytes), glucose: 4.6 mmol/L, protein: 0.36 g/L	LVEF of 15% with global hypokinesis	NR	PLEX 5 rounds over 11 days	6 days: improved EF of 55%; 2 weeks: weaned off pressors, extubated; 3 weeks: ambulating
11[8]	82/F	"Partial conduction block of the right ulnar nerve, peroneal nerve with dispersion, and tibial nerve with dispersion. Ulnar F waves prolonged"	"Consistent with GBS"	LVEF of 30%, "extensive apical akinesis"	ST elevation	PLEX x 5 sessions	2 weeks: improved strength; LVEF of 7%
12[16]	41/F	"NCS revealed a predominantly sensory axonal pattern of neuropathy affecting the arms and legs"	Protein count: 0.45 g/L	Apical ventricular dilation with overall mild to moderately impaired systolic function, as well as mildly impaired relaxation	ST elevation in leads V2–4 and ST depression in leads I and aVL	2 g/kg of IVIg over 3 days	6 months: no neuro symptoms and echo with improved systolic function (EF of 46%) and regional wall motion in the apical segments
Patient 1	58/M	Severe sensorimotor polyneuropathy with axonal and demyelinating features and marked conduction slowing	Cell count: 2/mm^3^; protein count: 93 mg/dl	Grade 1 diastolic dysfunction, EF of 15%, mild apical-lateral wall hypokinesis, and mild to moderate anterior wall hypokinesis	Sinus tachycardia with low voltage QRS. Later with T wave abnormality in inferolateral leads	0.4 g/kg daily IVIG x 7 days total	2 weeks: 2D echo normalized; EF >65%
Patient 2	79/F	Diffuse severe length-dependent sensorimotor axonal polyneuropathy	Cell count: 10/mm^3^; protein count: 54 mg/dl	EF of 30-35%, moderate-severe hypokinesis of the mid-LV segments with hyperdynamic basal and apical segments. RVSP of 35-39 mmHg	Sinus tachycardia, possible left atrial enlargement, left axis deviation, anterolateral infarct age undetermined	0.4 g/kg daily IVIG x 6 days total	6 days from initial echo with normal LVEF and mid-inferoseptal and inferior wall mild hypokinesis. Tracheostomy at discharge

## Conclusions

TCM should be recognized as a potential complication in GBS/AIDP patients with dysautonomia and should be managed in an ICU setting. With these cases, we add to the sparse body of literature describing the association between TCM and GBS/AIDP. It is not clear if female patients or if cases (like ours) with axonal variants of GBS are more predisposed to developing TCM; further, larger case series may help identify risk factors associated with the condition. We hope to raise awareness about this possible association in order to expedite the diagnosis and management of TCM in GBS patients with dysautonomia, and we propose a low threshold for TTE screening in the appropriate setting.

## References

[1] Willison HJ, Jacobs BC, van Doorn PA (2016). Guillain-Barré syndrome. Lancet.

[2] Dimachkie MM, Barohn RJ (2013). Guillain-Barré syndrome and variants. Neurol Clin.

[3] Boon M, Dennesen PJ, Veldkamp RF (2016). A rare stress cardiomyopathy in a patient with Guillain-Barré syndrome. Neth J Med.

[4] Zaeem Z, Siddiqi ZA, Zochodne DW (2019). Autonomic involvement in Guillain-Barré syndrome: an update. Clin Auton Res.

[5] Ramos A, Barbaran MA, Trujillo NE (2017). Takotsubo cardiomyopathy as a sequel of severe Dysautonomia from Guillain-Barré syndrome. J Neurol Sci.

[6] Fontenette R, Moses C, Rahman O (2013). Takotsubo cardiomyopathy associated with Guillain-Barre syndrome. Chest.

[7] Chakraborty T, Kramer CL, Wijdicks EFM, Rabinstein AA (2020). Dysautonomia in Guillain-Barré syndrome: prevalence, clinical spectrum, and outcomes. Neurocrit Care.

[8] Chandrasekaran PN, Pandey A, Idiculla PS (2020). Neuromuscular emergencies in the neuroscience intensive care unit. Neuromuscular Urgencies and Emergencies.

[9] Fugate JE, Wijdicks EF, Kumar G, Rabinstein AA (2009). One thing leads to another: GBS complicated by PRES and Takotsubo cardiomyopathy. Neurocrit Care.

[10] Gill D, Ruiz VG, Dean R, Liu K (2017). Takotsubo cardiomyopathy with Guillain-Barré syndrome. Proc (Bayl Univ Med Cent).

[11] Gravos A, Destounis A, Katsifa K (2019). Reversible stress cardiomyopathy in Guillain-Barré syndrome: a case report. J Med Case Rep.

[12] Kang CH, Oh JH, Song SK, Kang SY (2015). Takotsubo cardiomyopathy associated with Guillain-Barré syndrome. Korean J Clin Neurophysiol.

[13] Magid-Bernstein J, Al-Mufti F, Merkler AE (2016). Unexpected rapid improvement and neurogenic stunned myocardium in a patient with acute motor axonal neuropathy: a case report and literature review. J Clin Neuromuscul Dis.

[14] Martins RP, Barbarot N, Coquerel N, Baruteau AE, Kolev I, Vérin M (2010). Takotsubo cardiomyopathy associated with Guillain-Barré syndrome: a differential diagnosis from dysautonomia not to be missed. J Neurol Sci.

[15] Quick S, Quick C, Schneider R, Sveric K, Katzke S, Strasser RH, Ibrahim K (2013). Guillain-Barré syndrome and catecholamine therapy. A potential risk for developing takotsubo cardiomyopathy?. Int J Cardiol.

[16] Takemoto M, Yamashita T, Ohta Y (2018). Fulminant Guillain-Barré syndrome with Takotsubo cardiomyopathy: report of an autopsied case. Neurol Clin Neurosci.

[17] Jones T, Umaskanth N, De Boisanger J, Penn H (2020). Guillain-Barré syndrome complicated by takotsubo cardiomyopathy: an under-recognised association. BMJ Case Rep.

[18] Gill D, Liu K (2017). Takotsubo cardiomyopathy associated with Miller-Fisher syndrome. Am J Emerg Med.

